# Hydrogen Peroxide and Its Utility

**Published:** 1886-04

**Authors:** G. W. Smith

**Affiliations:** Cincinnati


### Hydrogen Peroxide and its Utility.

BY G. W. SMITH, CINCINNATI.

(Read before the Mississippi Valley Association.)

I DESIRE, in this paper, to call your attention to an agent of remarkable power and utility; one which, by virtue of its varied qualities, is foreshadowing the possibilities that are soon to be realized from its use.

Hydrogen Peroxide was discovered in 1818 by Thenard, a French chemist, and it was introduced to the medical profession in 1858 by Dr. Richard.

Passing over the chemistry of its production, it will be sufficient for the purpose of this article to merely state that it is liquid oxygen, and that the pure article gives off 475 volumes of oxygen. This agent contains a much larger constituent of oxygen than hydrogen, according to chemical test.

A pure article of liquid oxygen has the capacity of yielding up 475 volumes of oxygen; that is, when liberated, the gas will pass off in the form of oxygen, 475 times the volume of the water in which it was retained. It should be kept cool, quiet, and well corked, as the oxygen begins to escape at a temperature of about 60, or when the liquid is agitated. Its retaining solution is sulphuric acid.

We are told that the only caution necessary in the use of a good article, is, if at any time it causes irritation when applied internally, or locally, it is only necessary to add barium water, drop by drop, till turbidity ceases, then filter, if for immediate use; otherwise it may be allowed to precipitate; then carefully decant the supernatant liquid, leaving the milky deposit as refuse.

Ozone is the controlling principle in hydrogen peroxide, and liquid oxygen is the allotropic form of oxygen. Although but little is known of ozone, it has considerable interest attached to it in a sanitary point of view, and the extent of its presence in the air is registered at the Meteorological Observatories; and it is also embraced in the reports of the medical officers of the Government.

Ozone acts as a safeguard or regulator of the air. It is the active principle of oxygen. Remove it and all animal life would perish. And, upon the other hand, if it be present in excess it would prove alike destructive. It is not only the life-sustaining principle of oxygen, but it is also an agent capable of destroying infectious and fetid odors. It has a decided sanitary influence upon all disease-bearing and putrid matter which determine epidermic and endemic diseases.

Hydrogen Peroxide, when brought in contact with diseased germs, destroys their vitality, arrests further decomposition in organic matter, and restores the injured parts to a normal condition. It is cleansing, odorless, colorless, stimulating, and does not stain. It destroys pus, causes no pain by its application, is not poisonous, and is healing. The moment liquid oxygen touches pus, effervescence is the result, and this will continue until the last vestige of pus is absolutely destroyed. It may be applied to the most delicate tissue without pain or destruction.

Judging from the investigations and testimony of many eminent French and German writers, chemists and physicians, it may yet prove to be a formidable weapon in the hands of the dental profession, to battle with the various local diseases which present themselves for treatment in our daily practice. My own experience within the past year has convinced me that it is the most reliable anti-putrid application known, and if intelligently employed, it is as bland as water.

I wish to call your attention to a few practical tests in which I found it active, and efficient:

My first experience with hydrogen peroxide was in the treatment of a chronic alveolar abscess, involving the second superior bicuspid. According to the patient's own account, the fistulous opening had never ceased to discharge its vitiated contents for two years.

Leaving the tooth filled upon the anterior and posterior approximal surfaces, I approached the pulp-chamber by way of the grinding surface. After removing as far as possible, the debris from the root-canal, I formed a piston out of a suitable instrument, armed with cotton, and with this I succeeded in forcing the liquid oxygen through the root-canal. I repeated the operation, changing the cotton each time until it gave evidence upon the surface that the agent had completely penetrated the fistulous opening. I then introduced a pledget of cotton saturated with liquid oxygen, and sealed the opening with wax, and dismissed the patient with instructions to call for another application in three days. The patient called at the appointed time, when, to my great surprise the abscess had completely yielded to the first application. And at the point where the pus had previously found relief, there was a well defined cicatrix. I however made a second application and sealed the cavity as before, and at the expiration of seven days completed the operation by filling the root-canal. I have seen the patient frequently since, and up to the present time the tooth has remained perfectly healthy.

The second case was that of a first superior bicuspid, for which I had prepared to mount a porcelain crown. In the effort to enlarge the root-canal sufficiently to receive the pivot, I closed the upper portion of the canal, consequently there was no escape for the confined gas, and abscess was the result. I succeeded in relieving the pus sack at the apex of the root by opening up the root-canal. To this case I made three applications at intervals of three days. At the expiration of nine days all symptoms of alveolar abscess had subsided. I then proceeded to fill the upper third of the root-canal, and mounted the crown, which has been doing good service as a substitute for eight months.

The third case I have selected, from the many that I have treated with this agent, was a lower second biscupid. The tooth, upon the anterior approximal surface, contained a large gold filling, which doubtless was the primary cause of the devitalizing of the pulp. This case was one of no little interest. The abscess would make itself manifest about once in thirty days, more or less. The pus would, after its accumulation, gravitate to the surface and relieve itself, with but little pain or suffering to the patient. The fistulous opening would then close, leaving the periosteum, in that region thick and indurated. I gained access to the pulp chambers through the grinding surface and commenced the treatment with hydrogen peroxide. After the third or fourth application, the thickened and somewhat indurated condition of the periosteum began to grow less, and finally disappeared. The root-canal of this tooth has been filled six months without the least symptom of the former lesion.

I might had I the time and space, add many more cases of alveolar abscess that have yielded more promptly to this agent than to any antiseptic I have ever used.

And here, if you will allow the digression, I wish to record a prescription that has been well tested, and will be found invaluable as a dressing for root-canals. It may be introduced immediately before filling, or it may be applied to the root-canal and allowed to remain a day or two before filling.

℞ Iodoform, gr. xxv.
Bals. Peru, gtts. x.
Carvol, gtts. x.
Ungt. Petrolie, ℥ ss.
Rub well together.

The action liquid oxygen upon disease germs and morbific matter is, in appearance much like the culinary operation of whipping eggs. It has a decided advantage over many other antiseptics in that it not only destroys pus and bacteria, but dislodges decomposing matter, and imparts to the injured tissues new life and vigor.

Hydrogen peroxide possesses a power for bleaching which is seldom found in any agent, the principle of which is due to the ozone it contains. It may be applied with favorable results as a lotion, in the strength of ℥j to ℥iij of water, for mercurial stomatitis, foul and indolent ulcers, apthæ, and gangrene of the mouth; and also as a deodorizer in those very offensive conditions of the breath, caused by cheesy, granular, inspissated deposits about the tonsils. For we are convinced that in many cases this foul condition of the breath emanates from this often overlooked cause.

It will be found invaluable in the treatment of Riggs' disease, especially where the gums have become detached from the teeth, leaving deep pockets which are the receptacle of vitiated pulpy deposits, a cesspool of putrefaction, which may be completely controlled by this agent.

It may be applied in full strength for alveolar abscess, bleaching teeth, and as a dressing for all pus-discharging wounds: for lotions ℥j to ℥iij of water, and for internal use a two per cent. solution is sufficient.

As hydrogen peroxide is not a new remedy, I can not find words more appropriate to close this article than those written many years ago by Dr. Benjamin Rush: "Perhaps all the discoveries of future ages will consist more in new applications of established principles, and in new modes of exhibiting old medicines, than in the discovery of new theories, or of new articles of the Materia Medica."

